# The trajectory of functional status among older adults with chronic diseases and the association with social relationships

**DOI:** 10.3389/fpubh.2025.1492489

**Published:** 2025-04-07

**Authors:** Dandan Jiao, Yantong Zhu, Zhu Zhu, Xiang Li, Jinrui Zhang, Mingyu Cui, Yang Liu, Munenori Matsumoto, Yuko Sawada, Kumi Watanabe Miura, Taeko Watanabe, Tokie Anme

**Affiliations:** ^1^Department of Nursing, The First Affiliated Hospital and College of Clinical Medicine of Henan University of Science and Technology, Luoyang, China; ^2^Graduate School of Comprehensive Human Sciences, University of Tsukuba, Tsukuba, Japan; ^3^Faculty of Educational Science, Anhui Normal University, Wuhu, China; ^4^School of Public Health and Nursing, Hangzhou Normal University, Hangzhou, Zhejiang, China; ^5^College of Child Development and Education, Zhejiang Normal University, Hangzhou, Zhejiang, China; ^6^Department of Physical Therapy, Morinomiya University of Medical Sciences, Osaka, Japan; ^7^RIKEN Center for Advanced Intelligence Project, Nihonbashi, Tokyo, Japan; ^8^Faculty of Nursing, Shukutoku University, Chiba, Japan; ^9^Faculty of Medicine, University of Tsukuba, Tsukuba, Ibaraki, Japan

**Keywords:** chronic disease, functional status, older adults, social relationships, trajectory

## Abstract

**Background:**

Functional status crucially affects healthy aging. Identifying trajectory of functional status and related determinants is important. We aimed to identify the trajectory of functional status over 6 years among older adults with chronic diseases and its association with social relationships.

**Methods:**

A latent class growth model analysis was conducted to explore the trajectory of functional status using three time points over 6 years. After identifying the trajectories, a multi-nominal logistic regression analysis was performed to examine the association between social relationships and the trajectories.

**Results:**

We included data from 458 participants aged ≥65 years with chronic diseases in Japan. Three distinct trajectories were identified, with 73.3% of participants being functionally stable, 16.6% moderate functional decline, and 10.1% rapid functional decline. Good social relationships were associated with a lower probability of having moderate or rapid functional decline trajectories (odds ratio [OR] 0.75, 95% confidence interval [CI] 0.65–0.87 and OR 0.63, 95% CI 0.51–0.78, respectively).

**Conclusion:**

Differences were found in the functional trajectories of older adults with chronic disease over time. Older adults with more extensive social relationships appeared less likely to have a poorer functional trajectory. The findings suggest that fostering extensive social relationships could be an effective management strategy for functional decline deterioration.

## 1 Introduction

Functional status is a core indicator in relation to healthy aging, which involves older adults developing and maintaining an effective functional status enabling older adults to age healthily (1). Studies concerning functional ability have recently increased as impaired functional ability can result in adverse health outcomes, including cognitive impairment (2) and death (3). Effective interventional strategies are needed to address functional disability as it increases the global disease burden and health expenditure (4). Older people with chronic diseases have been reported to show earlier and more rapid functional decline compared with those without (5). Moreover, given the synergistic effects of chronic diseases and functional limitations on health outcomes (6), it is important to identify factors contributing to improved functional status, especially among older adults with chronic illness. Further, the World Health Organization has put forward proposals for achieving healthy aging, including identifying the trajectories and determinants of functional ability in relevant populations (7).

The Disablement Process Model (DPM), developed by Verbrugge and Jette (8), offers a framework for understanding how chronic conditions lead progressively from pathology to impairments, functional limitations, and ultimately, disability. The model highlights the role of external factors—such as personal resources and social relationships—in influencing this progression. Guided by the DPM, we conceptualize social relationships as a key determinant that may directly influence functional trajectories by promoting adaptive health behaviors, psychological well-being, and access to care-related resources (9). Specifically, we apply the model to formulate our research questions and select a person-centered approach—trajectory analysis—to identify distinct patterns of functional limitation over time. By linking these patterns with variations in social relationships, we aim to clarify their association with changes in functional status. This theoretical integration underpins both our rationale and methodological choices in examining functional change through the lens of the disablement process.

A 16-year longitudinal study conducted in Amsterdam involving adults aged ≥55 years reported three trajectories of functional limitation, namely, a high-level start and slight decline, a high-level start and late decline, and a low-level start and early-onset decline (10). Additionally, a population-based Korean study reported three trajectories of functional status (normal functioning, mild disability, and severe disability) (11). Another 20-year longitudinal study reported three functional trajectories: high stable, medium disability, and low disability at baseline followed by a slight decline (12). However, most of these studies focused on the general population; accordingly, changes in functional status among older adults with chronic diseases remain to be clarified. A nationwide longitudinal study conducted among older adults with disability reported five trajectories of physical frailty, namely, consistent frailty, consistent pre-frailty, worsening frailty, improving frailty, and consistently robust (13). However, as this study was based on nursing homes, its results may not be representative of community-based older adults. Since older adults with chronic disease have high incidence of functional limitations (14), knowing changes in functional status among them would provide an opportunity for intervention.

Japan’s aging population provides a critical context for studying functional trajectories. With more than 28.0% of its population aged 65 and older (15), Japan has the highest proportion of older adults globally. As the population ages and chronic disease prevalence rises, understanding functional status changes in older adults with chronic illnesses is essential for informing public health interventions. However, research on functional trajectories in this population remains limited, leaving gaps in understanding how chronic diseases contribute to functional decline and what factors may help influence these effects.

Relevant factors need to be identified that contribute to maintaining functional status or which slow functional decline. Regarding the determinants of functional trajectory, positive psychological well-being (12) and smoking cessation (11) have been associated with a latent functional trajectory over time. Social relationships benefit health outcomes and play an equally important role along with other well-established factors (e.g., a healthy diet) (9). The influence of social relationships on functional status could involve two possible mechanisms (16). First, social relationships promote positive behavior, for example, exercising as well as stopping smoking and consuming alcohol, which fosters good health. Second, from a psychological perspective, social relationships indirectly affect health outcomes through nurturing a sense of meaningfulness and purpose in life. However, the effects of social relationships on the functional trajectory over time have yet to be elucidated. One longitudinal study found that frequent social activities were linked to a decreased likelihood of having limited cognitive function (17). In contrast, another longitudinal study on aging reported that neither social isolation nor loneliness was associated with changes in frailty over an eight-year period (18). Additionally, a two-year population-based study reported that high social participation was associated with frailty improvement among frail older adults (19). However, that study only included data from two time points and used a variable-centered method, which may not have fully captured the functional trajectory of individuals. The variable-centered method identifies relationships among variables whereas a person-centered approach identifies similar patterns in the characteristics of individuals and is, therefore, more appropriate as individuals within a group are likely to have similarities and show different patterns of change over time (20). Growth mixture modeling is likely to be more effective in capturing differences in relation to individual changes (20).

We aimed to use a person-based growth mixture modeling method to explore functional changes among older adults with chronic diseases as well as the association between social relationships and the potential functional trajectories.

## 2 Materials and methods

### 2.1 Design and participants

This study utilized data from the Community Empowerment and Care for Well-being and Health Longevity Project, a longitudinal cohort study conducted in T Village, central Japan (21). This region is known for its flourishing agricultural industry. The project began in 1991 and has been conducted annually or biennially ever since. Its primary objective is to examine factors related to residents’ well-being in the context of low birth rates, rapid aging, and high medical expenditures. This study was conducted in collaboration with the T Municipality Government for health policy evaluation. To ensure community participation, all residents were informed about the survey, and the local government advertised it on the official website and sent detailed explanations to all households as part of the recruitment process. The relevant data were collected using self-administered questionnaires. Initially, questionnaires were distributed to all residents and collected after 2 weeks. Residents who required help completing the questionnaires were offered an interview to help complete their questionnaires. For this analysis, we extracted data from three time points: May 2011 (T1), May 2014 (T2), and May 2017 (T3), to explore factors related to functional trajectory and related factors.

Questionnaires were distributed to 1,085 participants. A total of 1,081 aged 65 and over responded to the survey in 2011 (T1). For the current study, we included participants with at least one chronic disease at the baseline year (T1) and we excluded participants with missing information regarding the main variables of functional status and index of social interactions (ISI) scores at the baseline year. The chronic disease include hypertension, stroke, heart disease, diabetes, hyperlipidemia, respiratory disease, muskuletical disease, cancer, immune disease, dementia, Parkinson’s disease, eye disorder, and ear disorder. Of the 1,081 respondents, 181 did not have chronic disease and 254 did not provide disease information. Initially, we selected 646 participants aged ≥65 years as targeted population. Among them, 64 participants without information regarding functional status and 124 participants without information on ISI scores were excluded. Finally, 458 participants (attrition rate 29.1%) who participated in all three waves were included and we collected data regarding their functional status in 2014 and 2017. A sensitivity analysis will compare the characteristics of the analyzed and excluded participants.

### 2.2 Functional status

Functional status was assessed using the Kihon checklist, which was developed by the Japan Ministry of Health, Labour, and Welfare. This checklist comprises seven categories evaluating daily lifestyle, physical strength, nutrition, oral health, home boundness, cognitive function, and depression mood (22). The daily lifestyle category includes five items of “Can you use the bus or train to go out by yourself?” “Do you go to buy daily necessary goods?” “Can you deal with financial issues (e.g., savings or deposits)?” “Do you go and visit your friends’ home?” “Do you advise to your friends or family members?” Physical strength category includes five items of “Can you go upstairs without using handrails or the wall?” “Can you stand up from a sitting position without grab anything?” “Can you continuously walk for 15 min?” “Have you experienced falls in the last year?” “Are you afraid of fall?” Nutrition category includes two items of “Does your weight decrease by 2–3 kg in the past 6 months?” and “Body mass index (Height: cm, Weight: kg).” Oral health includes three items of “Do you ever feel become difficulty in chewing tough foods than you did 6 months ago?” “Have you ever experienced choke or cough when drinking tea or soup?” “Do you feel thirsty or dry mouth?” Home boundness includes two items of “Do you go out at least once per week?” “Do you go out fewer times than you did in last year?” Cognitive function includes three items of “Do someone say you are forgetful or you always ask the same thing?” “Do you often search for the phone numbers by yourself and call someone?” “Do you occasionally not know what is the date today?”. Based on data availability, we used 20 items while excluding the depression mood category because our project questionnaire did not evaluate the depression category in T3. Each item was binary, offering a “yes” or “no” response, with one point assigned for each applicable “yes” answer. Total score of the 20 items was used, with a higher score indicated a worse functional status. Cronbach’s *α* values for the 20 items were 0.857, 0.858, and 0.850 for the three time points (T1, T2, and T3). The dimensionality and construct validity of the measure were tested using Confirmatory Factor Analysis (CFA). The CFA results indicated a good model fit for Time point 1 (CFI = 0.967, TLI = 0.960, RMSEA = 0.036), supporting the factor structure. Similarly, for time point 2, the model fit indices were CFI = 0.976, TLI = 0.970, and RMSEA = 0.034. For time point 3, CFA results also indicated a good model fit (CFI = 0.980, TLI = 0.975, RMSEA = 0.041). Factor loadings were all significant (*λ* > 0.50) across the three time points, further confirming construct validity. Additionally, reliability was assessed at each time point using composite reliability (CR ≥ 0.70) and average variance extracted (AVE ≥ 0.50).

### 2.3 Social relationships

Social relationships were evaluated using the ISI, which has been shown to have high validity and reliability among Japanese community residents, with a Cronbach’s *α* of 0.78 (23). In our study, Cronbach’s α was 0.77. The ISI evaluates five aspects through a total of 18 items: independence, social curiosity, interaction with others, social participation, and a feeling of safety. The Independence subscale consists of four items and assesses individuals’ motivation to live, adoption of an active approach in life, living with a regular lifestyle, and being motivated to live a healthy lifestyle. Social curiosity consists of five items and evaluates whether individuals read newspapers, read books, try the new equipment, have a hobby and a feeling of importance in society. Interaction, with three items, evaluates how individuals communicate with family and non-family members, as well as their interactions with the wider community. Social participation (four items) assesses individuals’ involvement in social groups, neighborhood activities, television viewing, and their active participation in society. Finally, the Feeling of Safety subscale (two items) examines whether individuals have access to counsel and support during emergencies. For each item, zero points were assigned when participants indicated that their engagement with these aspects was “rare,” and one point was assigned indicating that their engagement was “always,” “often,” or “sometimes.” The total score equalled the sum of each subscale score and ranged from 0 to 18, with higher scores indicating better social relationships.

### 2.4 Covariates

The questionnaires covered demographic characteristics, eating behavior, lifestyle habits, long-term care needs evaluation, evaluation of local services, social relationships, and medical conditions. Age, sex, exercise, and alcohol consumption/smoking, depression mood were covariates, with age treated as a continuous variable. Sex was coded as male and female. Regarding exercise, the respondents were asked “Do you exercise?” Responses of “always,” “frequently,” and “sometimes” were coded as “performing exercise” whereas responses of “no” were coded as “not exercising.” Regarding alcohol consumption, respondents were asked “Do you drink alcohol?” Responses of “always,” “everyday,” and “sometimes” were coded as “consumes alcohol” whereas responses of “hardly ever” and “do not consume alcohol” were coded as “non-consumers of alcohol.” For smoking, the respondents were asked “are you an active smoker?” Responses of “everyday” and “sometimes” were coded as “actively smoking” whereas responses of “I previously smoked but have now stopped” and “I do not smoke” were coded as “non-smoking behavior.” The depressive mood category assesses an individual’s mood over the past 2 weeks using five items: Do you feel that your life lacks fulfillment? Do you no longer enjoy activities you once did? Are tasks that were once easy now difficult? Do you feel that you are not a useful person? Do you feel exhausted without an apparent reason? Each item is scored as 1 point for “yes” and 0 points for “no.” The total depression score ranges from 0 to 5, with higher scores indicating greater levels of depression.

### 2.5 Statistical analysis

First, we determined the growth model change for the overall sample with non-growth, linear growth, and non-linear growth models considered in relation to the overall trajectory. We used a latent growth curve model to determine the overall change of functional status in the non-growth, linear, and latent basis models, sequentially. In the non-growth model, we assumed that there would not be changes in functional status over time. The intercept indicating the initial functional status of the participants was included in the model. In the linear growth model, we assumed a linear growth change in the functional status over time. In this model, we included the intercept representing the initial functional status and the slope representing the rates of change in functional status. In the latent basis model, we assumed that there was non-linear growth based on estimating the slope of time point 2 (T2). Several indices were employed to assess the model fit. The model showed a non-significant chi-square value (*χ*^2^). Moreover, comparative fit index (CFI) and Tucker–Lewis index (TLI) values were >0.90, while the root mean square error of approximation (RMSEA) and the standardized root mean square residual (SRMR) values were <0.08 (24). The Satorra–Bentler scaled chi-square test was then used to compare the fitted models (25). Maximum likelihood estimation with robust standard errors was used to deal with missing data in Mplus.

The number of classes was determined using the latent class growth model, which is a special growth mixture model type that assumes all individuals’ growth trajectories within one class have no variation (26). For model selection, we considered several model fit indicators in terms of Akaike information criteria (AIC), Bayesian information criteria (BIC), adjusted BIC (aBIC), Lo–Mendell–Rubin likelihood ratio test (LMR) values, and bootstrap likelihood ratio test (BLRT) values (27). Smaller AIC, BIC, and aBIC values indicate a better model fit (28). The LMR and BLRT were used to assess whether the k class was better than the *k*−1 class. Additionally, entropy was used for class selection, ranging from 0 to 1, with a higher score indicating better model goodness of fit.

Finally, we performed a multinomial logistic regression analysis to examine the predictive effect of ISI on class trajectories and included other baseline covariates in the model. The results are presented based on the Guidelines for Reporting on Latent Trajectory Studies (29).

Sensitive analysis was conducted to compare the demographic information between the analyzed participants and the excluded participants.

### 2.6 Ethical

This study was approved by the ethic committee of XXXX (No. 1331–3). The need for written informed consent was waived by the ethic committee due to retrospective nature of the study. According to the Japanese Ethical Guidelines for Medical and Health Research Involving Human Subjects, participants’ written informed consent was waived in this study because anonymous data were provided by the study area and participants reserved their right to opt out from the research. The study was performed in line with the Ethical Principles for Medical research Involving Human Subjects stated in the Declaration of Helsinki.

## 3 Results

Table 1 shows the baseline characteristic of participants. Among them (*n* = 458), the mean age at T1 was 74.9 years (standard deviation [SD] 6.9). Most participants were women, performed exercise, and were non-smokers/non-consumers of alcohol.

**Table 1:** Baseline characteristics of the participants.

Variables	Category	*n* (%)
Age (mean ± SD)		74.9 ± 6.9
Sex	Men	211 (46.1)
	Women	247 (53.9)
Living arrangements	Alone	16 (3.5)
	With others	438 (95.6)
	Missing data	4 (0.9)
Exercise	Yes	260 (56.8)
	No	169 (36.9)
Current alcohol consumer	Yes	79 (17.2)
	No	374 (81.7)
	Missing data	5 (1.1)
Current smoker	Smoker	39 (8.5)
	Non-smoker	399 (87.1)
	Missing data	30 (4.4)
Chronic disease (mean ± SD)		1.5 ± 0.8
Depression mood (mean ± SD)		0.6 ± 1.4
Social relationship (mean ± SD)		15.8 ± 2.4
T1 functional status (mean ± SD)		3.7 ± 4.0
T2 functional status (mean ± SD)		4.3 ± 4.2
T3 functional status (mean ± SD)		5.1 ± 4.1

The overall trajectory was determined using the non-growth, linear growth, and non-linear growth models. The non-growth model showed a poor model fit (*χ*^2^ = 107.610, *p* < 0.001, CFI = 0.408, TLI = 0.556, RMSEA = 0.238, SRMR = 0.182). The linear growth model fitted the data well (*χ*^2^ = 1.185, *p* = 0.276, CFI = 0.999, TLI = 0.997, RMSEA = 0.020, and SRMR = 0.013), whereas the non-linear growth model did not. The linear growth model showed significantly better fit than the non-growth model after applying the Satorra–Bentler chi-square test (*χ*^2^ = 119.943, *p* < 0.001). Thus, the linear growth model was selected. An intercept of 3.394 (*p* < 0.001) and an average slope of 0.475 (*p* < 0.001) showed that there were variations in the baseline functional status and limited change in functional status over time.

Table 2 presents model fit information. The two-class (Class 2), three-class (Class 3), and four-class (Class 4) models had significant LMR and BLRT values, indicating better model fit compared to the five-class (Class 5) and six-class (Class 6) models. However, one category in the Class 4 model included only 20 participants, falling below the recommended threshold of at least 5% (*n* = 22) of the total sample size for latent classes (30). Model selection was also based on AIC, BIC, and aBIC, where lower values indicate a better fit. Given these criteria, the Class 3 model was preferred over the Class 2 model. Ultimately, the Class 3 model was selected as the best-fitting model. The proportions of individuals in Classes 1, 2, and 3 were 10.1% (*n* = 43), 16.6% (*n* = 74), and 73.3% (*n* = 341), respectively.

**Table 2:** Model fit information.

Model	AIC	BIC	aBIC	Entropy	LMR	BLRT
Class 1	5379.244	5399.878	5384.010	1.0		
Class 2	4885.302	4918.317	4892.927	0.931	<0.001	<0.001
Class 3	4749.490	4794.885	4759.975	0.909	0.018	<0.001
Class 4	4677.381	4735.157	4690.725	0.913	0.043	<0.001
Class 5	4643.8788	4713.945	4659.992	0.907	0.111	<0.001
Class 6	4613.610	4696.148	4632.674	0.871	0.159	<0.001

AIC, Akaike information criteria; BIC, Bayesian information criteria; aBIC, adjusted Bayesian information criterion; BLRT, bootstrap likelihood ratio test; LMR, Lo–Mendell–Rubin likelihood ratio test.

Table 3 shows the bivariate analysis among the study covariates and trajectory groups. Supplementary Table 1 shows the results of univariate regression analyses examining the associations between baseline characteristic and functional status trajectory.

**Table 3:** Bivariate analysis between baseline characteristics and the trajectory groups of physical function.

Variables	Category	Functional stable	Moderate functional decline	Rapid functional decline	*χ^2^/Z*	*p*
		*n* (%)	*n* (%)	*n* (%)		
Age (mean ± SD)		72.7 ± 5.8	77.7 ± 6.9	84.0 ± 6.0	82.908	<0.001
Sex	Male	169 (51.7)	31 (39.7)	9 (28.1)	8.968	0.011
	Female	158 (48.3)	47 (60.3)	23 (71.9)		
Performs exercise	Yes	228 (69.7)	32 (41.0)	11 (34.4)	33.217	<0.001
	No	99 (30.3)	46 (59.0)	21 (65.6)		
Smoking and alcohol consumption	Yes	88 (27.2)	13 (16.9)	5 (16.1)	13.944	<0.001
	No	235 (72.8)	64 (83.1)	26 (83.9)		
Depression mood		0.3 ± 0.8	1.4 ± 1.9	2.7 ± 2.0	87.459	<0.001
Chronic disease (mean ± SD)		1.7 ± 1.0	2.1 ± 1.3	3.1 ± 1.9	25.382	<0.001
ISI		16.6 ± 1.8	14.6 ± 2.6	12.4 ± 2.9	94.550	<0.001

ISI, index of social interaction.

**Figure 1:**
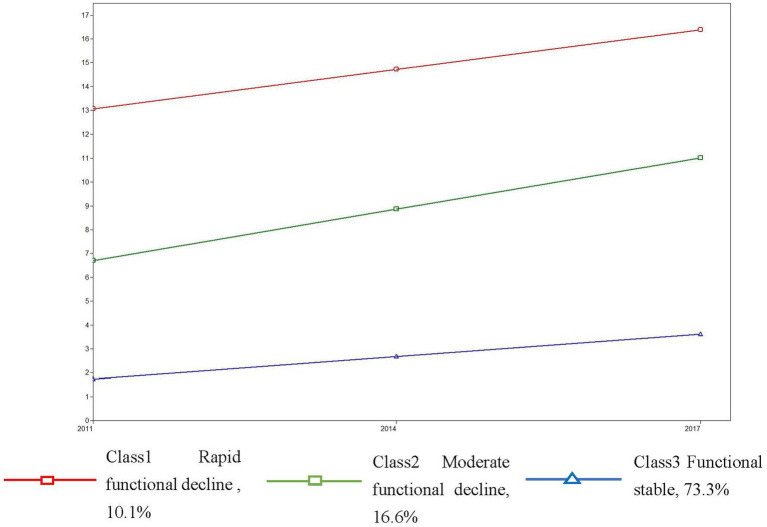
Class trajectory of the functional status.

Figure 1 shows class trajectory in terms of years. Class 1, the “rapid functional decline” class, had an average score of 6.707 (*p* < 0.001) for initial functional status and showed an increasing trend throughout the time period (slope, 2.154; *p* < 0.001). Class 2, the “moderate functional decline” class, had an average score of 13.066 (*p* < 0.001) for initial functional status, and showed a continuous increase (slope, 1.653; *p* < 0.05). Class 3, the “functional stable” class, had an average score of 1.741 (*p* < 0.001) for initial functional status, which showed a slight increase compared with class 2 (slope, 0.931; *p* < 0.001).

Table 4 shows the relationship between baseline ISI scores and functional status trajectory. Class 3 (functional stable) was taken as a reference group. Compared with functional stable class, higher ISI scores were associated with a lower likelihood (OR 0.75, 95% CI 0.65–0.89) of being in moderate functional decline or rapid function decline (OR 0.63, 95% CI 0.51–0.78). Compared with moderate functional decline class, higher ISI scores were associated with a lower likelihood of being in the rapid functional decline class (OR 0.83, 95% CI 0.69–0.91).

**Table 4:** Relationship between baseline ISI scores and trajectory groups in terms of functional status after adjusting for baseline characteristics.

Items	Moderate functional decline vs. Functional stable				Rapid functional decline vs. Functional stable				Rapid functional decline vs. Moderate functional decline			
	OR	95% CI			OR	95% CI			OR	95% CI		
ISI	0.75**	0.65	–	0.87	0.63**	0.51	–	0.78	0.83*	0.69	–	0.91
Age	1.15**	1.09	–	1.21	1.36**	1.23	–	1.50	1.17**	1.07	–	1.28
Sex	0.70	0.35	–	1.39	0.46	0.13	–	1.58	0.65	0.20	–	2.13
Performing exercise	0.34*	0.18	–	0.64	0.37	0.12	–	1.18	0.71*	0.37	–	3.34
Smoking/Alcohol consumption	1.15	0.49	–	2.73	1.22	0.26	–	1.18	1.11	0.32	–	6.13
Depression mood	1.92**	1.52	–	2.43	2.43	1.74	–	3.39	1.27	0.97	–	1.66

**p < 0.01, *p < 0.05.

CI, confidence interval; ISI, index of social interaction; OR, odds ratio.

ISI, and age were analyzed as continuous variables. The referred groups for sex, exercise, and alcohol consumption/smoking were men, not exercising, and no alcohol consumption/smoking.

Supplementary Table 2 shows the comparision between the analyzed participants (*n* = 458) and the excluded participants (*n* = 188) revealing statistically significant differences in age and sex (*p* < 0.001). The analyzed participants were younger (74.9 ± 6.9 vs. 78.8 ± 7.7) and more male (77.3% vs. 22.7%). No difference on the disease information (*p* = 0.781).

## 4 Discussion

This study examined the trajectories of functional status among older adults with chronic diseases over a six-year period. Multinomial regression analysis indicated that a high ISI score was associated with a reduced likelihood of being in rapid or moderate functional decline classes.

Our study contributes to the limited literature on functional status among older adults with chronic diseases. The trajectory classification in our study aligns with previous evidence, primarily derived from general older adult populations. A longitudinal study on older Americans of Mexican ethnicity used the Short Physical Performance Battery (SPPB), an objective measure of lower extremity function, to classify physical performance trajectories as stable, low decline, and high decline over 9 years (31). Similarly, a Japanese study used the Tokyo Metropolitan Institute of Gerontology Index of Competence (TMIG-IC), a measure of higher-level functional capacity, to classify trajectories as stable, low decline, and middle decline over 10 years (32). Meanwhile, Jonkman et al. (33) used self-reported Activities of Daily Living (ADL) and Instrumental ADL (IADL) to define functional trajectories of no decline, intermediate decline, and severe decline. Kihon Checklist (KCL) used in our study assesses multiple dimensions of health, including physical function, cognitive ability, mental well-being aspects, providing a broader perspective, which aligns with modern multidimensional aging models that integrate physical and cognitive health. Despite differences in measurement tools, all these studies reported a high proportion of individuals with relative functional stability, which ranged from 40.0 to 65.0%. In our study, 73.3% of the participants were in a stable function trajectory. This may have been due to our relatively short follow-up period (6 years) compared with >9 years in the other studies, as age is an important predictor of functional status. Nonetheless, our findings suggest that even older adults with chronic diseases may maintain functional stability or experience only gradual decline. This aligns with the DPM model, which conceptualizes disablement not as an inevitable, linear progression but as a dynamic process influenced by individual and contextual factors, including chronic illness and external resources such as social relationships. By identifying distinct functional trajectories in a high-risk group, our findings directly address our research question and underscore the utility of the DPM in capturing heterogeneity in the disablement process. Identifying functional trajectories could help guide chronic disease management and early interventions. Although the KCL provides a broader assessment of functional decline, particularly for early detection of multidimensional health risks, its composite nature may limit direct comparability with studies focused solely on physical function. Future research should compare different functional assessment tools, examining their sensitivity in detecting decline and their impact on healthcare decision-making. Additionally, studies should assess whether the choice of assessment tool influences long-term patient outcomes.

Our findings support the protective role of social relationships in functional aging. We found that a high ISI score was associated with a decreased likelihood of presenting with poorer trajectories, indicating that improved social relationships could have protective effects on functional status over time, which accord with the findings of a previous Canadian study (34). Additionally, increased social activities have been associated with an increased likelihood of being in a low disability trajectory group (35). Further, a greater number of social activities has been associated with a decreased likelihood of being in a low cognitive function trajectory group (15). According to the DPM, external factors such as social relationships may influence the progression from functional limitation to disability. Our findings offer empirical support for this theoretical pathway, reinforcing our hypothesis that social relationships are a key determinant of functional trajectories in older adults with chronic diseases. These results suggest that interventions aimed at enhancing social relationships—such as community-based programs, social participation initiatives, and policies that promote social connectedness—could be cost-effective strategies for maintaining functional health at a population level.

Given the strong association between social relationships and functional status, integrating social participation strategies into chronic disease management programs could help maintain functional independence in older adults. At the public health and policy level, local governments and community organizations should develop structured social support programs, such as peer support groups and virtual platforms, to foster connections among older adults, particularly those with chronic conditions. Additionally, policymakers should focus on creating age-friendly environments by ensuring accessible transportation, providing community spaces for social interaction, and offering incentives for organizations to support older adult volunteering opportunities. Future research should explore which types of social relationships offer the strongest protective effects and conduct multi-center or nationally representative studies to validate the generalizability of these findings across different healthcare systems and cultural contexts.

This study addresses a critical research gap by examining functional status trajectories in older adults with chronic diseases-an area that has received limited attention. While most previous studies have focused on functional aging in general older populations, our findings provide specific insights into a high-risk group. By applying growth mixture modeling, we captured the heterogeneity in functional changes over time, allowing for a more precise classification of functional subgroups. Understanding these trajectories and associated factors can help identify appropriate intervention targets to prevent functional decline in older adults with chronic diseases.

Despite its contribution, this study has several limitations. First, the change of participants disease during follow up was not in consideration because the chronic disease is a relative steady situation during 6 years. Second, the analyzed participants were older than the excluded cases that may have certain information bias, however, their disease condition were not different. Therefore, we believe our results can representative the changes of functional status of chronic disease population. Although the chronic disease profiles of included and excluded participants were comparable, differences in age could still affect functional status trajectories and limit the applicability of our findings to younger individuals with chronic illnesses. Third, our analysis indicated that missing data were not missing at random, suggesting that certain characteristics were associated with a higher likelihood of missing information. This introduces a potential selection bias, thereby limiting the generalizability of our findings to individuals with more stable health and stronger social relationships. Fourth, some key socioeconomic characteristics (income, assets, education, and employment status) were not included as covariates in our analysis due to cultural sensitivities and privacy concerns. As a result, these variables were omitted from the survey design to ensure participant comfort and response accuracy.

Socioeconomic status is a known determinant of both health outcomes and social relationships. For instance, individuals with higher income or education levels may have better access to healthcare, healthier lifestyles, or more extensive social networks, which could influence both social support and functional status. The absence of these covariates may thus affect the internal validity of our findings. Future studies should aim to incorporate these variables in a culturally sensitive manner to better assess their influence. Finally, as this study was based on a single regional dataset and not nationally representative data, caution should be exercised when generalizing our findings. Larger, multi-center, or nationally representative studies are needed to confirm these results and ensure their applicability across diverse populations and healthcare contexts.

## 5 Conclusion

Our findings showed that the trajectory of functional status among older adults was heterogeneous over time and was associated with social relationships. Promoting social relationships could improve the quality of life among older adults with chronic diseases.

## Data Availability

The data used in this study are available on reasonable request from the corresponding author if permission from the local municipal government of the study area is granted.

## Supplementary material

The Supplementary material for this article can befound online at: https://www.frontiersin.org/articles/10.3389/fpubh.2025.1492489/full#supplementary-material
