# Supplementary material for: The trajectory of functional status among older adults with chronic diseases and the association with social relationships
Source: Front Public Health. 2025 Apr 7;13:1492489. doi: 10.3389/fpubh.2025.1492489 (PMC12009918; doi:10.3389/fpubh.2025.1492489)
Supplement: Supplementary file 1 [file Table_1.docx]

Table 1 Bivariate analyses between baseline characteristic and trajectory groups in terms of functional status after adjusting for baseline characteristics

| Items | Moderate functional decline vs. Functional stable | | | |  | Rapid functional decline vs. Functional stable | | | |
| --- | --- | --- | --- | --- | --- | --- | --- | --- | --- |
|  | OR | 95% CI | | |  | OR | 95% CI | | |
| ISI | 0.66** | 0.58 | - | 0.75 |  | 0.52** | 0.45 | - | 0.61 |
| Age | 1.08** | 1.04 | - | 1.13 |  | 1.24** | 1.15 | - | 1.33 |
| Sex (reference: ) | 0.62 | 0.37 | - | 1.02 |  | 0.37 | 0.16 | - | 0.82 |
| Performingexercise(reference: none) | 0.30* | 0.18 | - | 0.50 |  | 0.23 | 0.11 | - | 0.49 |
| Smoking/Alcohol consumption | 1.84 | 0.97 | - | 3.50 |  | 9.94 | 0.93 | - | 79.88 |
| Depression | 1.92** | 1.58 | - | 2.34 |  | 2.65 | 2.08 | - | 3.39 |

**P<0.01, * P<0.05

ISI, index of social interaction

Table2 Baseline characteristic comparison between included and excluded participants

| Variables |  | Included  participants（n=458） | Excluded participant（n=188） | *χ^2^*/Z | *P* |
| --- | --- | --- | --- | --- | --- |
| Age |  | 74.9±6.96 | 78.78±7.7 | 2.594 | 0.108 |
| Sex | Male | 211 | 62 | 9.361 | <0.01 |
|  | Female | 247 | 126 |  |  |
| Chronic disease | 1 | 288 | 120 | 3.221 | 0.781 |
|  | 2 | 118 | 41 |  |  |
|  | ≥3 | 52 | 27 |  |  |
